# Chromosome genome assembly and whole genome sequencing of 110 individuals of *Conogethes punctiferalis* (Guenée)

**DOI:** 10.1038/s41597-023-02730-x

**Published:** 2023-11-16

**Authors:** Bojia Gao, Yan Peng, Minghui Jin, Lei Zhang, Xiu Han, Chao Wu, He Yuan, Andongma Awawing, Fangqiang Zheng, Xiangdong Li, Yutao Xiao

**Affiliations:** 1 Shenzhen Branch, Guangdong Laboratory of Lingnan Modern Agriculture, Key Laboratory of Gene Editing Technologies (Hainan), Ministry of Agriculture and Rural Affairs, Agricultural Genomics Institute at Shenzhen, Chinese Academy of Agricultural Sciences, Shenzhen, 518120 China; 2 Taishan Academy of Forestry Sciences, Taian, 271000 China; 3 Lancaster Environment Centre, Lancaster University, Lancaster, LAI 4YQ United Kingdom; 4 College of Plant Protection, Shandong Agricultural University, Taian, 271018 China

**Keywords:** Agricultural genetics, Genetic variation

## Abstract

The yellow peach moth, *Conogethes punctiferalis*, is a highly polyphagous pest widespread in eastern and southern Asia. It demonstrates a unique ability to adapt to rotten host fruits and displays resistance to pathogenic microorganisms, including fungi. However, the lack of available genomic resources presents a challenge in comprehensively understanding the evolution of its innate immune genes. Here, we report a high-quality chromosome-level reference genome for *C. punctiferalis* utilizing PacBio HiFi sequencing and Hi-C technology. The genome assembly was 494 Mb in length with a contig N50 of 3.25 Mb. We successfully anchored 1,226 contigs to 31 pseudochromosomes. Our BUSCO analysis further demonstrated a gene coverage completeness of 96.3% in the genome assembly. Approximately 43% repeat sequences and 21,663 protein-coding genes were identified. In addition, we resequenced 110 *C. punctiferalis* individuals from east China, achieving an average coverage of 18.4 × and identifying 5.8 million high-quality SNPs. This work provides a crucial resource for understanding the evolutionary mechanism of *C. punctiferalis*’ innate immune system and will help in developing new antibacterial drugs.

## Background & Summary

The yellow peach moth, *Conogethes punctiferalis* (Guenée) (Lepidoptera: Crambidae) is a polyphagous pest widely distributed in eastern and southern Asia, and can damage many crops, fruits, vegetables and spices^1^. *Ostrinia furnacalis* (Guenée) is the most important maize pest in most of Asia^2^. However, in some areas, *C*. *punctiferalis* has replaced and caused even more serious damage than *O*. *furnacalis* in recent years due to an enhanced fitness on maize^3^. It is challenging to control *C*. *punctiferalis* as its larvae bores into fruit and maize plants, enabling them to evade insecticide applications, entomopathogenic fungi and parasitoids^4^. *C. punctiferalis* infestations inflict serious damage to crops, and despite poor plant health, larvae survive well on moldy maize (Figure S1)^1^. Interestingly, recent studies showed that *C. punctiferalis* prefers to oviposit on apples infected with the fungus *Penicillium citrinum*, which may be explained by the altering the emission of host plant volatile organic compounds^5^. The bacteria *Wolbachia* can infect 66% of insect species and the infection rates follow a ‘most or few’ pattern, either being very high (>90%) or very low (<10%)^6^. In *C*. *punctiferalis*, the infection rate is only 4.5% which suggests that *C*. *punctiferalis* has the ability to resist some intracellular bacterial infections. In addition, few entomopathogenic fungi have been isolated from *C*. *punctiferalis* in its natural environment^7^. All of this suggest that, *C*. *punctiferalis* can adapt to a diverse microbial environment throughout its life history, which poses challenges for its management using entomopathogenic fungus. Genome analysis could provide valuable insights in developing pest control strategies for managing agricultural pests. The lack of a reference genome could hinder the understanding of molecular mechanisms underlying innate immune system. In this study, we constructed a chromosome-level genome assembly of *C*. *punctiferalis* using PacBio circular consensus sequencing (CCS) and Hi-C (High-throughput chromosome conformation capture) technology which provides a high resolution for phylogenetic and comparative genomic analyses. Furthermore, we have sequenced 110 *C*. *punctiferalis* individuals from 9 provinces in eastern China and generated a variants dataset containing 5.8 million SNPs which can be directly analysed without the need for re-processing the original data. Our study will facilitate the understanding of innate immune evolution of *C*. *punctiferalis* and help in developing new antibacterial drugs or an efficient pest control strategy.

## Methods

### Sample collection and sequencing

The experiment utilized a colony of *C*. *punctiferalis*, which has been maintained in the lab for more than 35 generations, originating from wild individuals collected from ears of maize in Tai’an, China (36° 10′ 00″ N, 117° 09′ 18″ E). The larvae used for sequencing were reared on fresh maize kernels at 25°C, a 14:10 (L:D) photoperiod cycle, and 75% relative humidity. A male and a female moth were chosen for mating. Among their offspring, a healthy male moth was selected for the extraction of the genomic DNA for PacBio HiFi sequencing. Additionally, another male individual was used for the construction of Hi-C library. The PacBio library (10 kb) was constructed and sequenced on Sequel II system. HiFi reads were produced from the raw subreads using the CCS workflow with the suggested parameters^8^. In total, it generated ~13.8 Gb PacBio long reads (N50 read length ~11.4 kb) which corresponded to approximately 28 × coverage of the genome. To achieve chromosome-level assembly, the Hi-C technique was used to identify contacts between different regions of the chromatin filament. The Hi-C library building process was carried out following the method described in a previous study^9^. A fifth instar male larvae from the same offspring was placed in formaldehyde to fix chromatin. The restriction enzyme *Dpn II* was used to digest DNA. The nucleotides of 5′ overhangs were labelled with biotin and blunt-end fragments were ligated. Subsequently, by reversing the crosslinks, the purified and sheared DNA was sequenced using a standard procedure on the Illumina platform with a paired-end (PE150) sequencing strategy.

Based on its distribution, a total of 110 *C*. *punctiferalis* individuals were sampled from nine locations in China: Anhui (n = 3), Hebei (n = 18), Henan (n = 14), Shandong (n = 50), Liaoning (n = 4), Jiangsu (n = 2), Sichuan (n = 3), Jiangxi (n = 1), and Hubei (n = 15) (Table S1). Genomic DNA (~0.4 μg per individual) was extracted from the whole body using DNeasy Blood and Tissue Kit (Qiagen, Hilden, Germany). For each individual, the genomic DNA was used to construct a sequencing library with an average insert size of approximately 350 bp. Subsequently, paired-end reads of 2 × 150 bp were generated using both the Illumina Novaseq 6000 platform and the BGISEQ-500 platform.

### Estimation of genome size and assembly of the genome

**Fig. 1:**
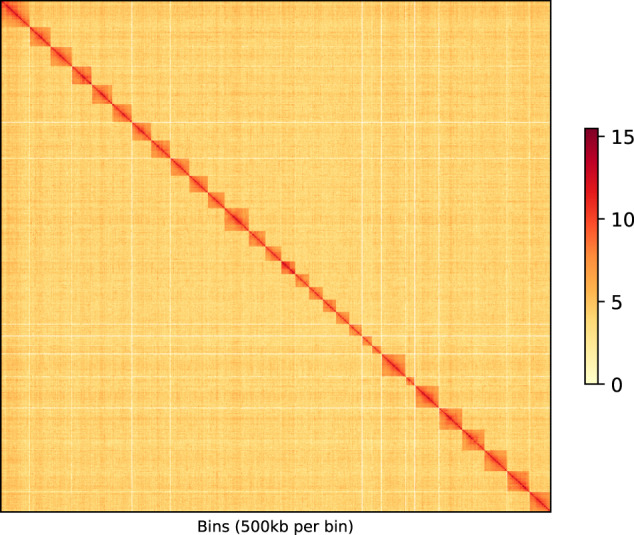
Genome-wide Hi-C intra-chromosome interactions in *C*. *punctiferalis* were visualized using a heat map. The interaction density was quantified based on the number of supporting Hi-C reads and represented by a colour bar, where dark red indicates high density and light pink indicates low density.

Genome size estimation, heterozygosity, and repetitiveness were analyzed using Jellyfish v2.2.10 and GenomeScope (http://qb.cshl.edu/genomescope/0) with 17 K-mer frequencies^10^, which predicted a genome size of approximately 440 Mb in homogametic males (Figure S2). The PacBio HiFi reads were used to produce final assemblies by Hifiasm v0.3.0 (https://github.com/chhylp123/hifiasm)^11^. Purge_Dups v1.2.3 (https://github.com/dfguan/purge_dups) was used to remove haplotigs^12^. The clean Hi-C generated reads were mapped to the draft assembly by BWA v0.7.5a^13^. Subsequently, the alignment file was used to produce scaffold-level assembly by SALSA2 (https://github.com/marbl/SALSA)^14^. Then the Hi-C generated reads mapped to scaffold-level assembly by BWA v0.7.5a and linkage information between scaffolds were saved as BAM files. Finally, the chromosome-level assembly was produced by ALLHiC v0.9.13 (https://github.com/tangerzhang/ALLHiC) with the parameters “-e GATC -k 31”^15^. BUSCO v4.0.0 was used to assess genome assembly with the insecta.odb10 database^16^. The assembled genome size was determined to be 494 Mb with a contig N50 of 3.25 Mb. Through Hi-C scaffolding, 1,226 out of 1,244 contigs were assigned to 31 pseudochromosomes, with an N50 of 17.97 Mb (Fig. 1, Table 1). In comparison to the GC content of Lepidoptera genomes, the *C*. *punctiferalis* genome exhibited a higher GC content at 39.5% (Table S2). Transposable elements (TEs) comprise approximately 43% of the *C*. *punctiferalis* genome. The most abundant repeat elements were long interspersed elements (LINEs), accounting for 16.95% (Table 2).

**Table 1:** Statistics of the *C*. *punctiferalis* genome assembly.

Features	
Genome size (Mb)	494
Karyotype	2n = 62
Number of contigs	1244
Number of assembled chrosomes	30 A + Z
Contig N50 (Mb)	3.25
Scaffold N50 (Mb)	17.97
BUSCO genes (%)	96.3
Repeat (%)	43.09
G + C (%)	39.50
Number of genes	21663

**Table 2:** Repetitive sequences in *C*. *punctiferalis* genome assembly.

Repeat class	Repeat subclass	number of elements	length occupied(bp)	Percentage of sequence
SINEs:		96094	14816960	3.00
	Penelope	931	135483	0.03
LINEs:		511926	83764690	16.95
	CRE/SLACS	562	142685	0.03
	L2/CR1/Rex	84203	16228622	3.28
	R1/LOA/Jockey	36601	9316751	1.89
	R2/R4/NeSL	778	433035	0.09
	RTE/Bov-B	295890	44041024	8.91
LTR		14994	6589811	1.33
	BEL/Pao	9085	2561754	0.52
	Ty1/Copia	1070	746301	0.15
	Gypsy/DIRS1	3259	2974438	0.60
DNA transposons		97452	20428101	4.13
	hobo-Activator	13589	2396344	0.48
	Tc1-IS630-Pogo	27473	5920118	1.20
	PiggyBac	2340	313997	0.06
	Tourist/Harbinger	444	72705	0.01
Rolling-circles		142454	26797809	5.42
Unclassified:		340721	55236734	11.18
Simple		90393	4917615	1.00
Low		8706	411704	0.08
Total bases masked			212963424	43.09

### Genome annotation

Prior to gene prediction, we employed RepeatModeler v1.0.11 (https://www.repeatmasker.org/RepeatModeler/) for *de novo* identification of repeat elements and used RepeatMasker v4.1.0 (http://www.repeatmasker.org) with the parameter “-e ncbi” to annotate the repeats in the genome sequence^17^. Then the BRAKER2 (https://github.com/Gaius-Augustus/BRAKER) pipeline was used to annotate the soft-masked genome with the parameters “--geneMarkGtf, --softmasking, --crf, --useexsiting”, which integrates RNAseq based, homology-based and *de novo* methods^18^. RNA was extracted from four developmental stages including eggs, fifth instar larvae (male and female), pupae (male and female), and adults (male and female) using Trizol and sample integrity was analysed on an Agilent 2100 Bioanalyzer (Agilent, Santa Clara, CA, USA). The construction of the cDNA library and paired-end RNA-seq (Illumina) were carried out by Grandomics Co. Ltd. The RNA-seq data of different developmental stages were mapped to genome by STAR v 2.7.1a (https://github.com/alexdobin/STAR) to generate RNA-Seq spliced alignment^19^. Meanwhile, protein sequences of six insect genomes (*Chilo suppressalis*, *Ostrinia furnacalis*, *Amyelois transitella*, *Galleria mellonella*, *Drosophila melanogaster*, *Bombyx mori*) obtained from NCBI were mapped to *C*. *punctiferalis* genome using GenomeThreader (https://genomethreader.org/) with the parameters “-gff3out, -skipalignmentout”. Then RNAseq and homologous proteins alignment information was used for GeneMark-ET v4 (http://exon.gatech.edu/GeneMark/license_download.cgi) and AUGUSTUS v3.3.3 (https://github.com/Gaius-Augustus/Augustus) training and gene prediction. Function annotation was carried out by aligning the predicted gene sets to NCBI-NR, Kyoto Encyclopedia of Genes and Genomes (KEGG) and eggNOG databases^20^. The GO annotation and InterPro and PFAM domain identification was obtained using InterProscan prediction^21^. Using the BRAKER2 genome annotation pipeline, a total of 21,663 protein-coding genes were annotated and the average lengths of CDS were 1,485 bp. The functional annotation showed that 99.35% of genes had hits in the Nr databases and 78.20% in the eggnog database (Table 3). Additional assembly and annotation statistics can be found in Tables 1, 3. The annotations of gene structure, gene function, and repeats were uploaded in figshare (see Data Records).

**Table 3:** Functional annotation of the *C*. *punctiferalis* genome assembly.

	Number	Percentage (%)
Total	21666	100
Nr	21525	99.35
KEGG	8428	38.90
eggNOG	16943	78.20
GO	9803	45.25
Interpro	14270	65.86

### Variants calling and filtering

**Fig. 2:**
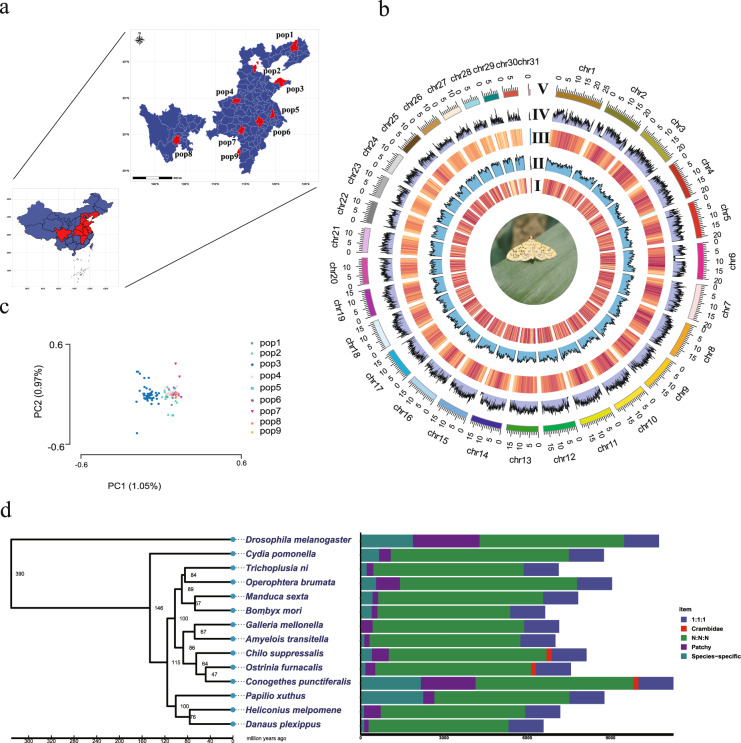
Population structure of wild-caught *C*. *punctiferalis* and genome evolution of *C*. *punctiferalis*. (**a**) Sampling locations of the *C*. *punctiferalis* analysed in this study. Pop1 - Liaoning: Shenyang, Pop2 - Hebei: Langfang, Pop3 - Shandong: Yantai, Pop4 - Henan: Zhengzhou, Pop5 - Jiangsu: Yangzhou, Pop6 - Anhui: Lujiang, Pop7 - Hubei: Wuhan, Pop8 - Sichuan: Qianwei, Pop9 – Jiangxi:Pingxiang. (**b**) Circular diagram depicting the genomic landscape of 31 *C*. *punctiferalis* chromosomes (Chr1 - Chr31 on a Mb scale). Track 1: Distribution of gene density with sliding windows of 200 kb. Track 2: Distribution of repeat sequence with sliding windows of 200 kb. Track 3: Distribution of GC content density with sliding windows of 200 kb. Track 4: Distribution of SNPs density with sliding windows of 50 kb. Track 5: 31 chromosomes of the *C*. *punctiferalis* chromosomes. (**c**) Population structure of 110 strains according to the first two principal components (PC1 and PC2). (**d**) Lepidopteran orthology across thirteen sequenced species. The number on each node indicates the divergent time. Bootstrap values based on 1000 replicates and all nodes received bootstrap support = 100 except one node (bootstrap support = 92). Bars are subdivided to represent different types of orthology clusters as indicated. Comparison of the gene repertoire of fourteen insect genomes. “1:1:1” indicates single-copy genes, “N:N:N” indicates orthologous genes present in multiple copies in all the fourteen species, “Crambidae” indicates common gene families unique to Crambidae, “patchy” indicates the existence of other orthologs that are presented in at least one genome.

**Fig. 3:**
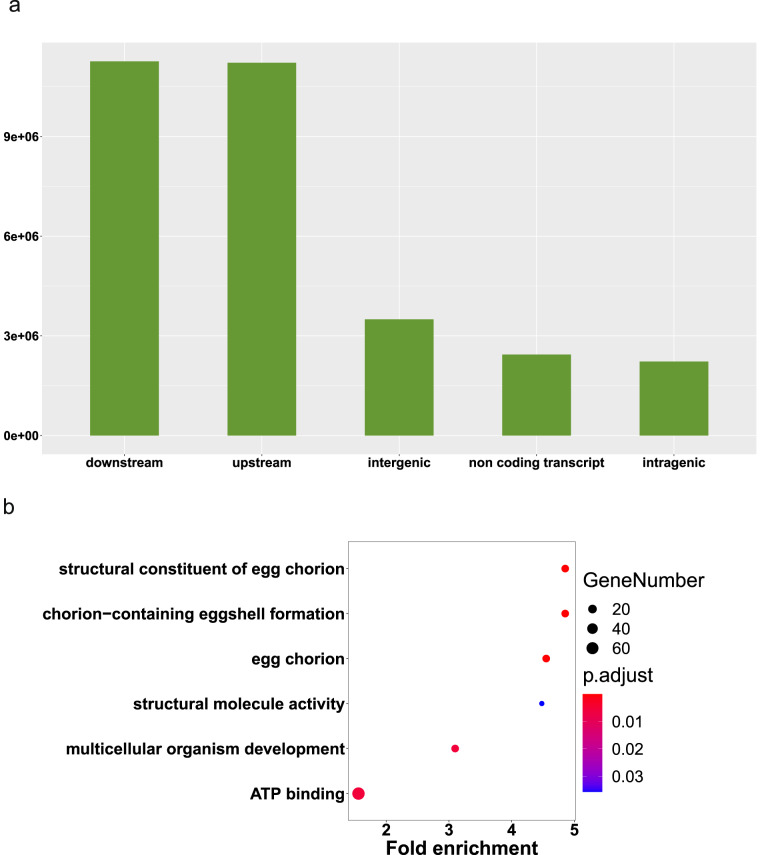
Summary of SNPs in *C*. *punctiferalis*. (**a**) The count of SNPs in different types. (**b**) GO enrichment analysis of genes with the lowest 5% *Π* score.

Quality control of sequencing data was conducted by fastp (https://github.com/OpenGene/fastp)^22^. The clean reads were mapped to the reference genome using BWA v0.7.5^13^. Variant calling was carried out by Genome Analysis Toolkit (GATK v 4.0.12.0)^23^. The PCR duplicates of each of the samples were marked by MarkDuplicates and HaplotypeCaller was run on each bam file to call SNPs. The SNPs were further filtered using the following criteria: “QD < 2.0 || FS > 60.0 || MQ < 40.0 || SOR > 3.0 || MQRankSum < −12.5 || ReadPosRankSum < −8.0”. SNPs with minor allele frequency (MAFs) less than 5% were discarded. The missing rate of a genotype for population was 0. Linkage disequilibrium pruning was performed by PLINK v1.90b6.24 using a window size of 50 SNPs (advancing 5 SNPs at a time) and an *r*^2^ threshold of 0.5^24^. The filtered SNPs sets were used for PCA performed by GCTA v1.93.2 (https://yanglab.westlake.edu.cn/software/gcta/#Download)^25^. The acquired SNPs were annotated and categorized by SnpEff (http://pcingola.github.io/SnpEff/download/)^26^. We sampled a total of 110 *C. punctiferalis* individuals from 9 provinces in eastern China and carried out genome sequencing (Fig. 2a). Each individual was sequenced at an average of ~18.4 × , yielding a total of 4104.9 Gb of data (Table S1). The analysis of the 5.8 million high-quality single nucleotide polymorphisms (SNPs) revealed that a majority of these SNPs are situated either upstream (36.04%) or downstream (36.17%) (Fig. 3a). The SNP density within the genome is shown in Fig. 2b and the average nucleotide diversity (*Π*) was 3 × 10^−3^. According to Gene Ontology (GO) enrichment analysis of genes with the lowest 5% *Π* score, these genes were enriched in molecular functions including egg chorion (GO:0042600), multicellular organism development (GO:0007275) and ATP binding (GO:0005524) (Fisher’s exact test, *p < *0.05) (Fig. 3b).

## Data Records

The CCS reads (SRR25434291), Hi-C reads (SRR25443395), RNA-Seq reads (SRR25435727, SRR25435728, SRR25435729 and SRR25435730), and resequencing data (SRR25472874 - SRR25472913 and SRR25619108 - SRR25619177) have been deposited in the NCBI’s Sequence Read Archive (SRA), under study accession number SRP451679^27^. The reference genome was deposited in the GenBank with the accession number GCA_031163375.1^28^. The genetic variation data have been deposited in the European Variation Archive (EVA) with accession number ERZ21819523^29^. The results of genome annotation, variants calling, orthology, genome synteny, and positive selection were deposited in the Figshare database^30^. For convenience, data are organized into fourteen files:

1. blastp_result.txt (2.88 MB): This file contains the annotation result from the alignment to NCBI-NR.
2. emapper.annotations (12.06 MB): This file contains the annotation result from the alignment to the eggNOG database. It also contains GO and KEGG terms.
3. interproscan.tsv (1019.61 MB): This file contains the annotation results from the alignment to multiple structure databases.
4. gene_structure_ypm.gff (121.11 MB): It provides various genomic features of the genome, such as gene and transcript locations and structure.
5. ypm_genome.codingseq (34.85 MB): This file contains predicted gene sets of the whole genome in FASTA format.
6. positive_selection.tsv (29.17 kB): This file contains information about genes that showed positive selection in the genome of *C*. *punctiferalis*.
7. C. punctiferalis-Cn. medinalis.collinearity (346.86 kB): This file contains identified syntenic blocks information between *C. punctiferalis* and *Cn. medinalis*.
8. C. punctiferalis-Ch. suppressalis.collinearity (350.41 kB): This file contains identified syntenic blocks information between *C. punctiferalis* and *Ch. suppressalis*.
9. Organism.tree (0.43 kB): It provides the phylogenetic tree of fourteen species in NEWICK format.
10. ortho.csv (1.59 kB): This file contains the number of orthologous genes present in different species.
11. go_enrich.csv (16.16 kB): It provides data from the GO enrichment analysis of expanded gene families in *C*. *punctiferalis* compared to the ancestor.
12. snp_count.csv (0.14 kB): It provides the count of SNPs based on their genomic locations.
13. low_pi_go_enrich.txt (15.92 kB): This file contains the list of genes with the lowest 5% *Π* score.
14. repeats.gff (121.11 MB): It provides genomic features of the repeats sequence identified in the genome.

## Technical Validation

### Genome assembly assessment

The completeness of genome assembly was assessed by searching for 1,367 single-copy insect genes using Benchmarking Universal Single-Copy Ortholog (BUSCO). The assembly contains 95.4% complete and single-copy BUSCOs, 0.8% complete and duplicated, 0.1% fragmented and 3.7% missing (Table 1). The Hi-C heatmap was used to confirm the accuracy of the chromosome assembly which revealed a well-organized interaction contact pattern along the diagonals within/around the chromosome inversion region (Fig. 1). Furthermore, we used minimap2 v 2.17-r954-dirty with the parameters “-ax map-pb” to align the assembly with the HiFi data^31^. A total of 99.87% of the HiFi reads were mapped to the genome assembly and the coverage rate was 99.83%.

### Orthology validation

The protein-coding sequences of Lepidoptera except for *C*. *punctiferalis* and *D*. *melanogaster* (FlyBase Release 6.32) were downloaded from NCBI and Ensembl. Each gene with the longest transcript was used to identify orthologous groups. Orthologous genes cluster were performed with OrthoFinder v2.4.0 (https://github.com/davidemms/OrthoFinder)^32^. Single-copy orthologous genes were extracted from the clustering results. Multiple alignments were independently performed for each group using MAFFT v7.453 with the “-auto” parameter and TrimAL v1.2rev59 was used for alignment trimming^33^. The alignments of all single-copy orthologs were concatenated to a super gene. A species tree was built using IQ-TREE v2.0.6 (https://github.com/Cibiv/IQ-TREE) with a model selection across each partition and 1000 ultrafast bootstrap replicates^34^. R8s v1.81 was used to estimate species divergence time and mean substitution rates^35^. Two secondary calibration points obtained from the TimeTree database were used to calibrate our phylogeny: 67 and 76–102 million years ago for the split time of *B*. *mori* – *M*. *sexta* and *D*. *plexippus* – *H*. *melpomene*, respectively^36^.

The phylogeny was analysed using 1,213 single-copy orthologs of 13 lepidopteran species and *D*. *melanogaster* as an outgroup. The phylogenetic analysis showed that the yellow peach moth *C*. *punctiferalis* and the Asian corn borer *O*. *furnacalis* formed a sister lineage to the Crambidae. The three Crambidae (*Ch*. *suppressalis*, *C*. *punctiferalis* and *O*. *furnacalis*) and two Pyralidae (*G*. *mellonella* and *A*. *transitella*) species were clustered together and diverged less than 100 Myr ago (Fig. 2d). There were 10,971 orthogroups (70.5% of 15,557 orthogroups) shared by Crambidae and Pyralidae (Fig. 2d). For the three Crambidae species, *C*. *punctiferalis* shares more orthologs with *O*. *furnacalis* than *Ch*. *suppressalis* (Figure S3).

**Fig. 4:**
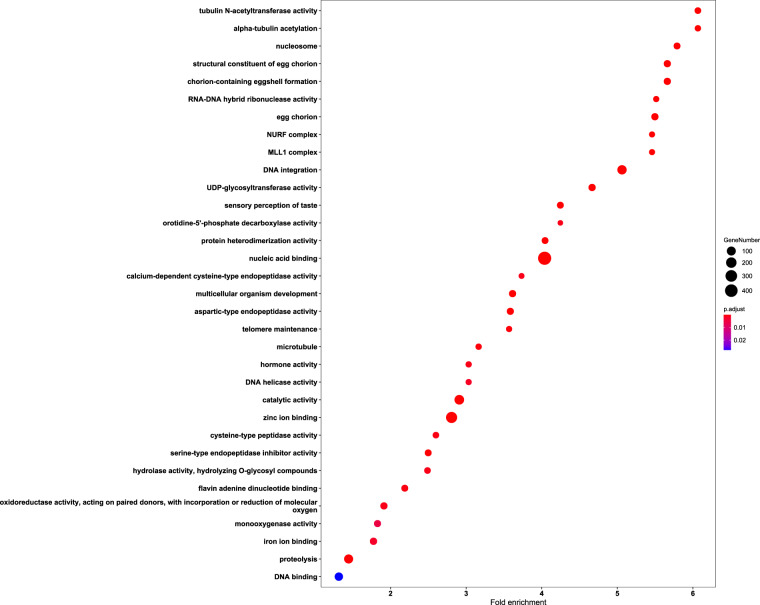
GO enrichment analysis of expanded gene families in *C*. *punctiferalis* compared to the ancestor.

The identification of each gene family expansions and contractions within the phylogeny was obtained using CAFÉ v 4.0.1 with a *p*-value < 0.05 as the cut-off^37^. Expanded gene set enrichment analysis was carried out using R package clusterProfiler^38^. 5,686 orthogroups are generally conserved across different species, and 2,167 ones are species-specific for *C*. *punctiferalis* (Fig. 2d). In comparison to the common ancestor of *C*. *punctiferalis* and *O*. *furnacalis*, there were 1,834 gene families that exhibited expansion, while 1,121 gene families experienced contraction in *C*. *punctiferalis*. The expansion genes were involved in 33 classes of molecular functions including the catalytic activity (GO:0003824) and zinc ion binding (GO:0008270) (Fisher’s exact test, *p < *0.05) (Fig. 4). From the analysis on the domain of expanded gene families, a majority of them were related to transposon activity and membrane proteins (Table S3).

### Genome synteny

The genome of *C*. *punctiferalis* was compared with the genomes of two other Crambidae species, *Ch*. *Suppressalis* and *Cnaphalocrocis medinalis*, respectively. Collinear blocks that included a minimum of five genes were identified using MCScanX^39^. The analysis revealed 413 syntenic blocks between *C*. *punctiferalis* and *Ch*. *Suppressalis*, and 304 syntenic blocks between *C*. *punctiferalis* and *Cn. medinalis*. In the genome of *C*. *punctiferalis*, there were 5,662 genes that exhibited relatively conservation and could be identified in both *C*. *punctiferalis*-*Ch*. *suppressalis* and *C*. *punctiferalis*-*Cn*. *medinalis* syntenic blocks. Out of the genes in *C*. *punctiferalis*, only 2,266 genes were found to be present in *C*. *punctiferalis*-*Ch*. *suppressalis* syntenic blocks, while 2,441 genes from *C*. *punctiferalis* were identified in *C*. *punctiferalis*-*Cn*. *medinalis* syntenic blocks. The identified syntenic blocks information have been uploaded to figshare (see Data Records).

### Positive selection

We tested positive selection on 5,584 single-copy genes of Pyraloidea lineages including three Crambidae species (*O*. *furnacalis*, *Ch*. *suppressalis* and *C*. *punctiferalis*) and two Pyralidae species (*A*. *transitella* and *G*. *mellonella*). PRANK v130410 and PAL2NAL v14 were used to align proteins and generate codon alignments^40^. The branch-site model aBSREL of HYPHY v2.5.28 framework (https://github.com/veg/hyphy) was used to analyse positive selection on *C*. *punctiferalis*^41^. The dn/ds less than 50 across more than 5% sites and *p*-value of less than 0.05 at *C*. *punctiferalis* node were kept. There were 154 genes showing the signature of positive selection in the genome of *C*. *punctiferalis*. The information regarding these positively selected genes has been uploaded to figshare (see Data Records).

### Population structure

The pairwise *F*_*ST*_ of all populations of *C*. *punctiferalis* were calculated using VCFtools v0.1.13 software with window size 10 kb^42^. The results of principal component analysis (PCA) and *F*_*ST*_ showed that there was no significant differentiation among different geographic populations (Fig. 2c and Table S4). Due to various reasons, we could not sample equal numbers of individuals from all locations. The uneven sampling might have an impact on the final results. However, the results from our population genetics studies are consistent with the results from a previous study^43^.

### Supplementary information

Chromosome genome assembly and whole genome sequencing of 110 individuals of Conogethes punctiferalis (Guenée)

## Data Availability

All software and pipelines were executed following the manuals and protocols provided by the published bioinformatic tools. The version and parameters of software have been described in Methods. If no detail parameters were mentioned for a software, default parameters were used as recommended by developer.
