# Supplementary material for: Chromosome genome assembly and whole genome sequencing of 110 individuals of Conogethes punctiferalis (Guenée)
Source: Sci Data. 2023 Nov 16;10:805. doi: 10.1038/s41597-023-02730-x (PMC10654572; doi:10.1038/s41597-023-02730-x)
Supplement: Supplementary file 1 — Chromosome genome assembly and whole genome sequencing of 110 individuals of Conogethes punctiferalis (Guenée) [file 41597_2023_2730_MOESM1_ESM.docx]

Supplementary Information

**Chromosome genome assembly and whole genome sequencing of 110 individuals of *Conogethes punctiferalis* (Guenée)**

Bojia Gao^1†^, Yan Peng^1†^, Minghui Jin^1†^, Lei Zhang^1†^, Xiu Han^2^, Chao Wu^1^, He Yuan^1^, Andongma Awawing^3^, Fangqiang Zheng^4^, Xiangdong Li^4^, Yutao Xiao^1^*

1. Shenzhen Branch, Guangdong Laboratory of Lingnan Modern Agriculture, Key Laboratory of Gene Editing Technologies (Hainan), Ministry of Agriculture and Rural Affairs, Agricultural Genomics Institute at Shenzhen, Chinese Academy of Agricultural Sciences, Shenzhen 518120, China

2. Taishan Academy of Forestry Sciences, Taian 271000, China

3. Lancaster Environment Centre, Lancaster University, Lancaster LAI 4YQ, United Kingdom.

4. College of Plant Protection, Shandong Agricultural University, Taian 271018, China

| Figure | Page |
| --- | --- |
| Figure S1 | 2 |
| Figure S2 | 3 |
| Figure S3 | 4 |


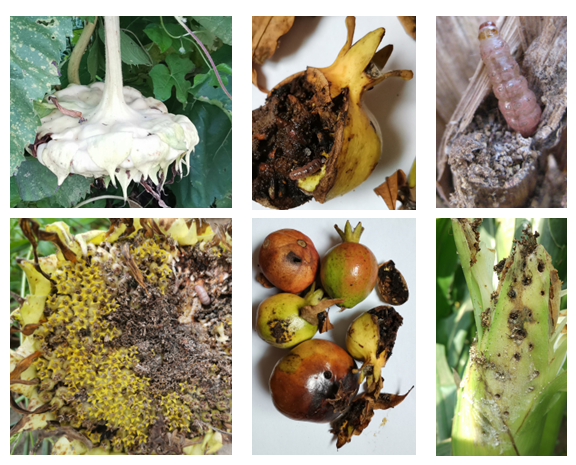


Figure S1. *C. punctiferalis* caused damage on three host. The first column is sunflower. The second column is pomegranate. The third column is maize.


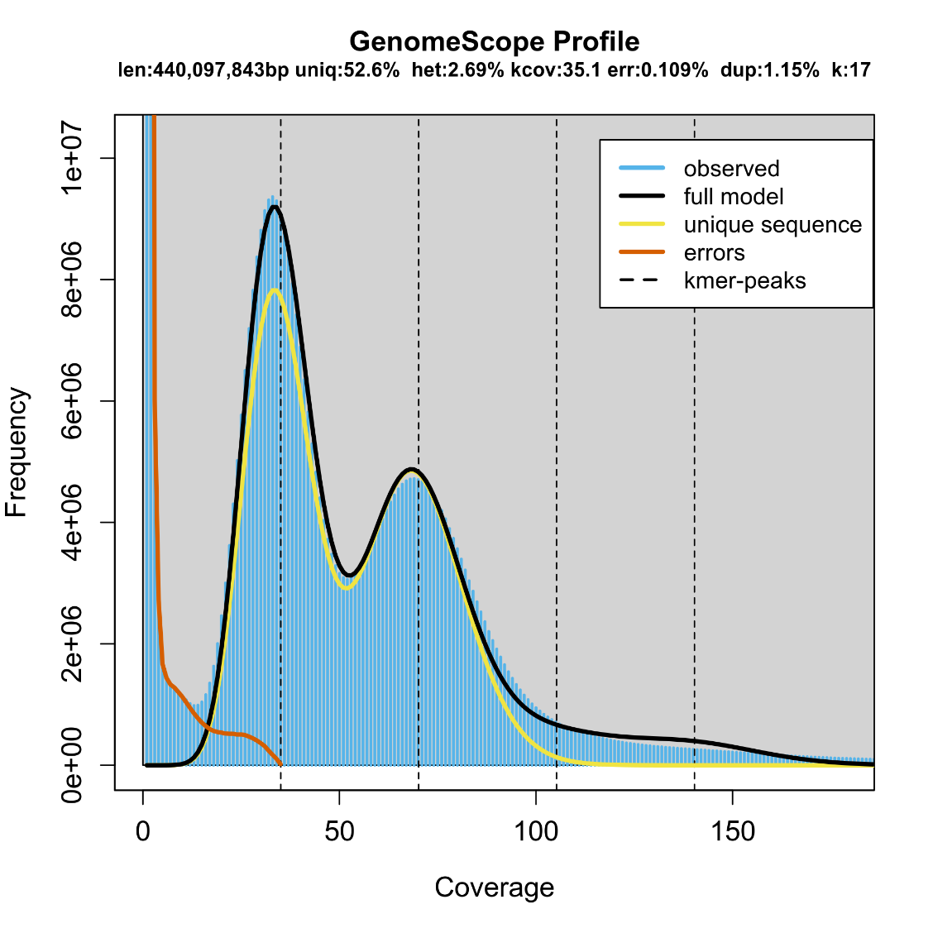


Figure S2. K-mer plot of the *C. punctiferalis* genome. K-mer plot showing the distribution of k-mer copy number (KCN) at 17-mer for the *C. punctiferalis* genome.


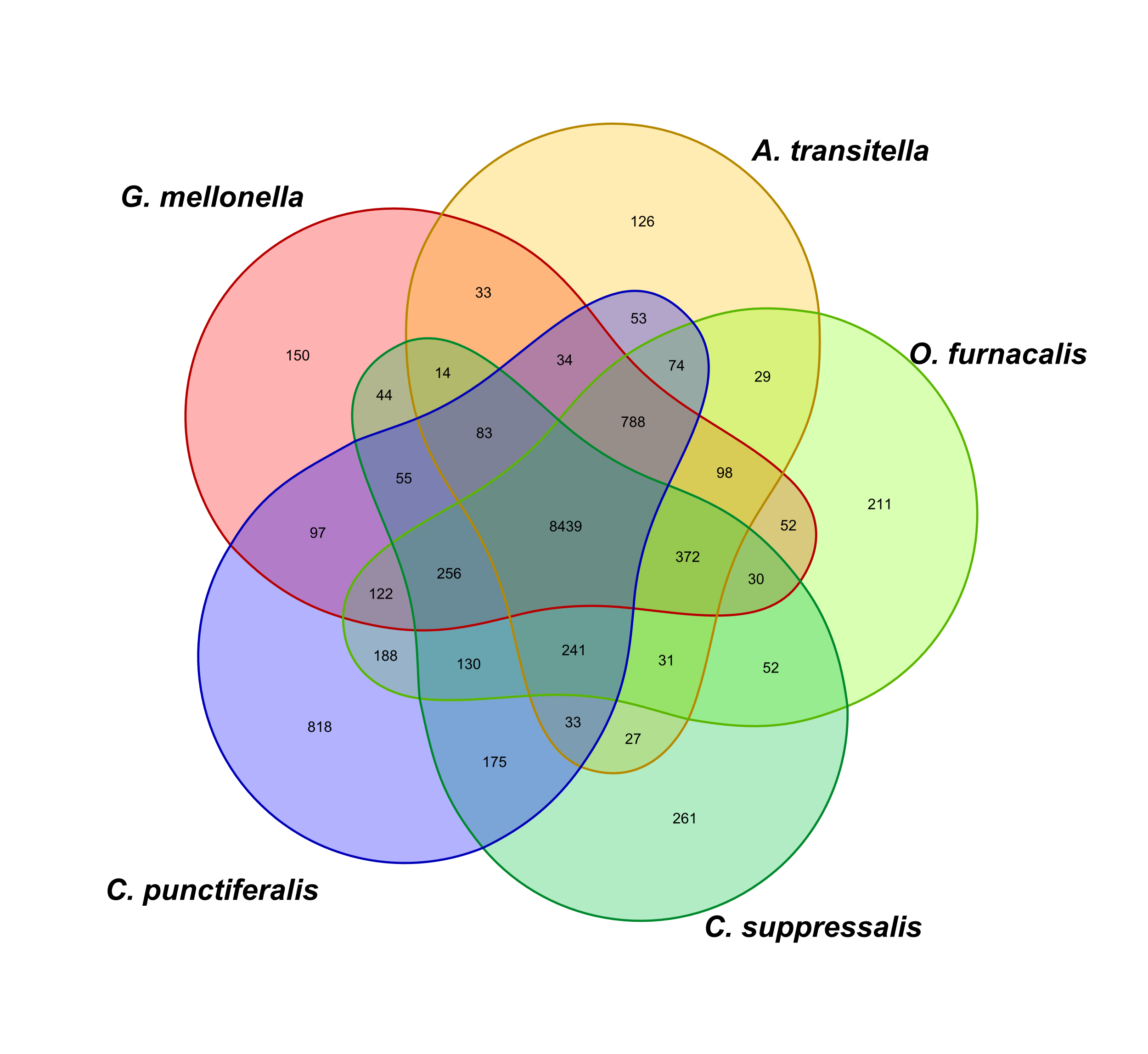


Figure S3. Venn diagram illustrating the unique and shared gene family clusters in five species.

Table S1. Summary of *C. punctiferalis* genome sequencing data

| Sample Identifier | Colllection date | Sampling Locations | Total data(Gb) | Depth |
| --- | --- | --- | --- | --- |
| AHLJ-15 | 2020-08-10 | Anhui:Lujiang(31.46°N,117.46°E) | 12.88389 | 26.08075 |
| AHLJ-17 | 2020-08-10 | Anhui:Lujiang(31.46°N,117.46°E) | 12.73069 | 25.77063 |
| AHLJ-19 | 2020-08-10 | Anhui:Lujiang(31.46°N,117.46°E) | 13.05247 | 26.42201 |
| HBDP1 | 2020-08-08 | Hubei:Wuhan(30.75°N,114.80°E) | 8.308037 | 16.81789 |
| HBDP10 | 2020-08-08 | Hubei:Wuhan(30.75°N,114.80°E) | 8.540246 | 17.28795 |
| HBDP3 | 2020-08-08 | Hubei:Wuhan(30.75°N,114.80°E) | 8.639336 | 17.48853 |
| HBDP4 | 2020-08-08 | Hubei:Wuhan(30.75°N,114.80°E) | 8.178011 | 16.55468 |
| HBDP5 | 2020-08-08 | Hubei:Wuhan(30.75°N,114.80°E) | 9.881869 | 20.00378 |
| HBDP6 | 2020-08-08 | Hubei:Wuhan(30.75°N,114.80°E) | 9.608756 | 19.45092 |
| HeB1 | 2020-08-04 | Hebei:Langfang(39.51°N,116.61°E) | 30.39854 | 61.5355 |
| HeB2 | 2020-08-04 | Hebei:Langfang(39.51°N,116.61°E) | 36.67008 | 74.23093 |
| HeB3 | 2020-08-04 | Hebei:Langfang(39.51°N,116.61°E) | 31.22875 | 63.21609 |
| HeB4 | 2020-08-04 | Hebei:Langfang(39.51°N,116.61°E) | 29.63353 | 59.9869 |
| HeB5 | 2020-08-04 | Hebei:Langfang(39.51°N,116.61°E) | 39.15923 | 79.26969 |
| HeB6 | 2020-08-04 | Hebei:Langfang(39.51°N,116.61°E) | 24.25713 | 49.10351 |
| HeB7 | 2020-08-04 | Hebei:Langfang(39.51°N,116.61°E) | 25.85642 | 52.34093 |
| HeB8 | 2020-08-04 | Hebei:Langfang(39.51°N,116.61°E) | 43.95631 | 88.98038 |
| HEDP1 | 2020-08-04 | Hebei:Langfang(39.51°N,116.61°E) | 8.428234 | 17.0612 |
| HEDP10 | 2020-08-04 | Hebei:Langfang(39.51°N,116.61°E) | 9.910037 | 20.0608 |
| HEDP2 | 2020-08-04 | Hebei:Langfang(39.51°N,116.61°E) | 8.554718 | 17.31724 |
| HEDP3 | 2020-08-04 | Hebei:Langfang(39.51°N,116.61°E) | 9.154721 | 18.53182 |
| HEDP4 | 2020-08-04 | Hebei:Langfang(39.51°N,116.61°E) | 8.762168 | 17.73718 |
| HEDP5 | 2020-08-04 | Hebei:Langfang(39.51°N,116.61°E) | 8.751489 | 17.71556 |
| HEDP6 | 2020-08-04 | Hebei:Langfang(39.51°N,116.61°E) | 9.20268 | 18.62891 |
| HEDP7 | 2020-08-04 | Hebei:Langfang(39.51°N,116.61°E) | 8.505033 | 17.21667 |
| HEDP8 | 2020-08-04 | Hebei:Langfang(39.51°N,116.61°E) | 8.222247 | 16.64423 |
| HEDP9 | 2020-08-04 | Hebei:Langfang(39.51°N,116.61°E) | 9.571304 | 19.37511 |
| HN1 | 2020-08-04 | Henan:Zhengzhou(34.91°N,114.05°E) | 28.86183 | 58.42476 |
| HN2 | 2020-08-04 | Henan:Zhengzhou(34.91°N,114.05°E) | 25.78609 | 52.19855 |
| HN5 | 2020-08-04 | Henan:Zhengzhou(34.91°N,114.05°E) | 36.987 | 74.87246 |
| HN6 | 2020-08-04 | Henan:Zhengzhou(34.91°N,114.05°E) | 28.8555 | 58.41194 |
| HN7 | 2020-08-04 | Henan:Zhengzhou(34.91°N,114.05°E) | 25.85661 | 52.34131 |
| HN8 | 2020-08-04 | Henan:Zhengzhou(34.91°N,114.05°E) | 23.72439 | 48.02508 |
| HNDP1 | 2020-08-04 | Henan:Zhengzhou(34.91°N,114.05°E) | 8.832193 | 17.87893 |
| HNDP10 | 2020-08-04 | Henan:Zhengzhou(34.91°N,114.05°E) | 8.950884 | 18.1192 |
| HNDP3 | 2020-08-04 | Henan:Zhengzhou(34.91°N,114.05°E) | 8.000295 | 16.19493 |
| HNDP4 | 2020-08-04 | Henan:Zhengzhou(34.91°N,114.05°E) | 8.963382 | 18.1445 |
| HNDP5 | 2020-08-04 | Henan:Zhengzhou(34.91°N,114.05°E) | 9.21251 | 18.6488 |
| HNDP6 | 2020-08-04 | Henan:Zhengzhou(34.91°N,114.05°E) | 9.028753 | 18.27683 |
| HNDP8 | 2020-08-04 | Henan:Zhengzhou(34.91°N,114.05°E) | 9.075766 | 18.372 |
| HNDP9 | 2020-08-04 | Henan:Zhengzhou(34.91°N,114.05°E) | 9.032585 | 18.28459 |
| HuB10 | 2020-08-08 | Hubei:Wuhan(30.75°N,114.80°E) | 24.46053 | 49.51524 |
| HuB11 | 2020-08-08 | Hubei:Wuhan(30.75°N,114.80°E) | 20.64986 | 41.80133 |
| HuB8 | 2020-08-08 | Hubei:Wuhan(30.75°N,114.80°E) | 16.98653 | 34.38569 |
| HuB9 | 2020-08-08 | Hubei:Wuhan(30.75°N,114.80°E) | 25.11043 | 50.83083 |
| JSSDP1 | 2020-08-11 | Jiangsu:Yangzhou(32.68°N,119.61°E) | 8.571089 | 17.35038 |
| JSSDP4 | 2020-08-11 | Jiangsu:Yangzhou(32.68°N,119.61°E) | 9.078279 | 18.37708 |
| LNDP1 | 2020-08-01 | Liaoning:Shenyang(41.83°N,123.57°E) | 8.547846 | 17.30333 |
| LNDP2 | 2020-08-01 | Liaoning:Shenyang(41.83°N,123.57°E) | 9.021981 | 18.26312 |
| LNSDP1 | 2020-08-01 | Liaoning:Shenyang(41.83°N,123.57°E) | 8.781534 | 17.77639 |
| LNSDP2 | 2020-08-01 | Liaoning:Shenyang(41.83°N,123.57°E) | 8.796606 | 17.80689 |
| PX-1 | 2020-08-19 | Jiangxi:Pingxiang(27.29°N,113.88°E) | 13.07248 | 26.46251 |
| SC-10 | 2020-08-21 | Sichuan:qianwei(29.11°N,104.03°E) | 11.99034 | 24.27195 |
| SC-17 | 2020-08-21 | Sichuan:qianwei(29.11°N,104.03°E) | 8.752409 | 17.71743 |
| SC-4 | 2020-08-21 | Sichuan:qianwei(29.11°N,104.03°E) | 11.95647 | 24.20338 |
| SD1 | 2020-08-14 | Shandong:Yantai(37.21°N,120.63°E) | 25.85051 | 52.32896 |
| SD10 | 2020-08-14 | Shandong:Yantai(37.21°N,120.63°E) | 19.45375 | 39.38006 |
| SD11 | 2020-08-14 | Shandong:Yantai(37.21°N,120.63°E) | 20.577 | 41.65384 |
| SD13 | 2020-08-14 | Shandong:Yantai(37.21°N,120.63°E) | 22.26258 | 45.06594 |
| SD14 | 2020-08-14 | Shandong:Yantai(37.21°N,120.63°E) | 27.39343 | 55.45228 |
| SD15 | 2020-08-14 | Shandong:Yantai(37.21°N,120.63°E) | 26.67284 | 53.99361 |
| SD18 | 2020-08-14 | Shandong:Yantai(37.21°N,120.63°E) | 18.22531 | 36.89334 |
| SD19 | 2020-08-14 | Shandong:Yantai(37.21°N,120.63°E) | 24.06851 | 48.72167 |
| SD2 | 2020-08-14 | Shandong:Yantai(37.21°N,120.63°E) | 24.43473 | 49.46302 |
| SD22 | 2020-08-14 | Shandong:Yantai(37.21°N,120.63°E) | 18.21695 | 36.87643 |
| SD23 | 2020-08-14 | Shandong:Yantai(37.21°N,120.63°E) | 24.28429 | 49.15848 |
| SD24 | 2020-08-14 | Shandong:Yantai(37.21°N,120.63°E) | 19.60205 | 39.68026 |
| SD26 | 2020-08-14 | Shandong:Yantai(37.21°N,120.63°E) | 25.72573 | 52.07638 |
| SD30 | 2020-08-14 | Shandong:Yantai(37.21°N,120.63°E) | 36.43273 | 73.75046 |
| SD32 | 2020-08-14 | Shandong:Yantai(37.21°N,120.63°E) | 27.23497 | 55.13151 |
| SD33 | 2020-08-14 | Shandong:Yantai(37.21°N,120.63°E) | 45.44403 | 91.99197 |
| SD35 | 2020-08-14 | Shandong:Yantai(37.21°N,120.63°E) | 47.01708 | 95.17627 |
| SD4 | 2020-08-14 | Shandong:Yantai(37.21°N,120.63°E) | 24.03211 | 48.648 |
| SD40 | 2020-08-14 | Shandong:Yantai(37.21°N,120.63°E) | 31.28888 | 63.33782 |
| SD41 | 2020-08-14 | Shandong:Yantai(37.21°N,120.63°E) | 31.28999 | 63.34005 |
| SD42 | 2020-08-14 | Shandong:Yantai(37.21°N,120.63°E) | 30.53686 | 61.81551 |
| SD45 | 2020-08-14 | Shandong:Yantai(37.21°N,120.63°E) | 38.19573 | 77.3193 |
| SD46 | 2020-08-14 | Shandong:Yantai(37.21°N,120.63°E) | 34.98502 | 70.81988 |
| SD47 | 2020-08-14 | Shandong:Yantai(37.21°N,120.63°E) | 32.51708 | 65.82406 |
| SD48 | 2020-08-14 | Shandong:Yantai(37.21°N,120.63°E) | 36.00204 | 72.87862 |
| SD5 | 2020-08-14 | Shandong:Yantai(37.21°N,120.63°E) | 22.50704 | 45.56081 |
| SD50 | 2020-08-14 | Shandong:Yantai(37.21°N,120.63°E) | 30.61195 | 61.9675 |
| SD52 | 2020-08-14 | Shandong:Yantai(37.21°N,120.63°E) | 35.5682 | 72.00041 |
| SD53 | 2020-08-14 | Shandong:Yantai(37.21°N,120.63°E) | 27.62422 | 55.91947 |
| SD6 | 2020-08-14 | Shandong:Yantai(37.21°N,120.63°E) | 32.27357 | 65.33112 |
| SD8 | 2020-08-14 | Shandong:Yantai(37.21°N,120.63°E) | 22.61165 | 45.77258 |
| SD9 | 2020-08-14 | Shandong:Yantai(37.21°N,120.63°E) | 21.9582 | 44.44979 |
| SDDP1 | 2020-08-14 | Shandong:Yantai(37.21°N,120.63°E) | 8.719542 | 17.65089 |
| SDDP10 | 2020-08-14 | Shandong:Yantai(37.21°N,120.63°E) | 9.738321 | 19.7132 |
| SDDP2 | 2020-08-14 | Shandong:Yantai(37.21°N,120.63°E) | 8.342244 | 16.88713 |
| SDDP3 | 2020-08-14 | Shandong:Yantai(37.21°N,120.63°E) | 8.361111 | 16.92533 |
| SDDP4 | 2020-08-14 | Shandong:Yantai(37.21°N,120.63°E) | 8.625629 | 17.46079 |
| SDDP5 | 2020-08-14 | Shandong:Yantai(37.21°N,120.63°E) | 8.441082 | 17.08721 |
| SDDP6 | 2020-08-14 | Shandong:Yantai(37.21°N,120.63°E) | 8.432318 | 17.06947 |
| SDDP7 | 2020-08-14 | Shandong:Yantai(37.21°N,120.63°E) | 9.503334 | 19.23752 |
| SDDP8 | 2020-08-14 | Shandong:Yantai(37.21°N,120.63°E) | 9.2649 | 18.75486 |
| SDDP9 | 2020-08-14 | Shandong:Yantai(37.21°N,120.63°E) | 7.997945 | 16.19017 |
| SHDDP1 | 2020-08-14 | Shandong:Yantai(37.21°N,120.63°E) | 8.279079 | 16.75927 |
| SHDDP10 | 2020-08-14 | Shandong:Yantai(37.21°N,120.63°E) | 8.563091 | 17.33419 |
| SHDDP4 | 2020-08-14 | Shandong:Yantai(37.21°N,120.63°E) | 8.844475 | 17.9038 |
| SHDDP5 | 2020-08-14 | Shandong:Yantai(37.21°N,120.63°E) | 8.570567 | 17.34933 |
| SHDDP6 | 2020-08-14 | Shandong:Yantai(37.21°N,120.63°E) | 8.411182 | 17.02668 |
| SHDDP7 | 2020-08-14 | Shandong:Yantai(37.21°N,120.63°E) | 8.723584 | 17.65908 |
| SHDDP8 | 2020-08-14 | Shandong:Yantai(37.21°N,120.63°E) | 8.894555 | 18.00517 |
| SHDDP9 | 2020-08-14 | Shandong:Yantai(37.21°N,120.63°E) | 8.724206 | 17.66034 |
| WH-26 | 2020-08-08 | Hubei:Wuhan(30.75°N,114.80°E) | 11.44138 | 23.16069 |
| WH-28 | 2020-08-08 | Hubei:Wuhan(30.75°N,114.80°E) | 18.00302 | 36.44335 |
| WH-46 | 2020-08-08 | Hubei:Wuhan(30.75°N,114.80°E) | 10.43365 | 21.12074 |
| WH-49 | 2020-08-08 | Hubei:Wuhan(30.75°N,114.80°E) | 18.95283 | 38.36606 |
| WH-50 | 2020-08-08 | Hubei:Wuhan(30.75°N,114.80°E) | 38.67821 | 78.29597 |

Table S2. Comparison of our *C. punctiferalis* genome GC content with other published genomes

| species | *B. mori* | *M. sexta* | *Ch. suppressalis* | *P. xylostella* | *S. frugiperda* | *D. plexippus* | *C. punctiferalis* |
| --- | --- | --- | --- | --- | --- | --- | --- |
| genome size (Mb) | 460.3 | 470 | 783.4 | 323.3 | 383.9 | 248.7 | 494 |
| number of chrosomes | 28 | 28 | 30 | 31 | 31 | 30 | 31 |
| number of contigs | 726 | 6,226 | 158 | 39 | 172 | 10,791 | 1244 |
| contig N50 (Mb) | 12.2 | 0.42 | 20.2 | 11 | 4.9 | 0.1 | 3.25 |
| Scaffold N50 (Mb) | 16.8 | 14.2 | 28.3 | 11.3 | 13 | 9.2 | 17.9 |
| G+C (%) | 38.5 | 36 | 37 | 38 | 36 | 32 | 39.5 |
| BUSCO genes (%) | 98.9 | 99 | 97.5 | 99 | 99.6 | 98.9 | 96.2 |
| number of genes | 17,047 | 19,909 | 11,366 | 16,283 | 14,679 | 14,669 | 21,663 |

Table S3. The number of identified conserved domains with specific expansion in the *C. punctiferalis*.

| **database** | **ID** | **annotation** | **counts** |
| --- | --- | --- | --- |
| Gene3D | G3DSA:2.40.70.10 | Acid Proteases | 15 |
| Phobius | SIGNAL_PEPTIDE_C_REGION | C-terminal region of a signal peptide. | 125 |
| SUPERFAMILY | SSF56672 | DNA/RNA polymerases | 62 |
| SUPERFAMILY | SSF56219 | DNase I-like | 59 |
| Phobius | SIGNAL_PEPTIDE_H_REGION | Hydrophobic region of a signal peptide. | 88 |
| ProSiteProfiles | PS50994 | Integrase catalytic domain profile. | 28 |
| Pfam | PF00665 | Integrase core domain | 13 |
| PANTHER | PTHR22955:SF60 | PROTEIN CBG26950 | 30 |
| Phobius | TRANSMEMBRANE | Region of a membrane-bound protein predicted to be embedded in the membrane. | 155 |
| Phobius | CYTOPLASMIC_DOMAIN | Region of a membrane-bound protein predicted to be outside the membrane, in the cytoplasm. | 91 |
| Phobius | NON_CYTOPLASMIC_DOMAIN | Region of a membrane-bound protein predicted to be outside the membrane, in the extracellular region. | 141 |
| Pfam | PF00078 | Reverse transcriptase (RNA-dependent DNA polymerase) | 52 |
| SUPERFAMILY | SSF53098 | Ribonuclease H-like | 54 |
| SignalP_GRAM_POSITIVE | SignalP-TM | SignalP-TM | 60 |
| Phobius | SIGNAL_PEPTIDE | Signal peptide region | 36 |
| SUPERFAMILY | SSF56672 | DNA/RNA polymerases | 62 |
| PANTHER | PTHR11505 | L1 TRANSPOSABLE ELEMENT-RELATED | 26 |
| Pfam | PF14223 | gag-polypeptide of LTR copia-type | 15 |

Table S4. Pairwise *F_ST_* values among the localities.

|  | Liaoning | Henan | Hebei | Hubei |
| --- | --- | --- | --- | --- |
| Shandong | 0.0035715 | 0.0015456 | 0.0013913 | 0.0020526 |
| Liaoning |  | 0.0022582 | 0.0019450 | 0.0013848 |
| Henan |  |  | 0.0003798 | 0.0002139 |
| Hebei |  |  |  | 0.0002811 |

note: The localities (Anhui, Sichuan, Jiangsu, Jiangxi) that amounts of samples less than four are not count
